# Frequency and factors associated with focused antenatal care in Guinea: Analysis of the DHS 2018

**DOI:** 10.4102/jphia.v15i1.505

**Published:** 2024-10-29

**Authors:** Maimouna Baldé, Jean B.D. Loua, Tiany Sidibé, Fanta Barry, Bienvenu S. Camara, Ramata Diallo, Madeleine Toure, Kaba S. Keita, Sadan Camara, Mamadou D. Balde

**Affiliations:** 1 Department of Public Health, Center for Research in Reproductive Health in Guinea, Conakry, Guinea; 2 Department of Public Health, Centre National de Formation et de Recherche en Santé Rural de Maferinyah, Forécariah, Guinea; 3 Department of Public Health, Faculty of Health Sciences and Techniques, Gamal Abdel Nasser University of Conakry, Conakry, Guinea

**Keywords:** focused antenatal care, frequency, factors associated, Demographic and Health Survey, Guinea

## Abstract

**Background:**

In Guinea, despite women’s recourse to antenatal care (ANC), little remains known about the use of focused antenatal care (FANC), contained in the ANC package.

**Aim:**

The aim of this study was to analyse the frequency and factors associated with FANC, using data from the Demographic and Health Survey (DHS) 2018.

**Setting:**

This study was conducted in Guinea.

**Methods:**

This was a secondary analysis of data from the DHS conducted in 2018 in Guinea. It included all women who achieved at least one ANC visit in the last 2 years prior to the survey. Multivariate logistic regression was received to identify factors associated with FANC. Stata 16 software was used for the analysis.

**Results:**

This study shows that in Guinea, between 2016 and 2018 only 33% of women undergoing ANC received a FANC. The most commonly used service was blood pressure measurement (93%), while the least commonly used service was deworming (42%). Factors associated with FANC were living in the Kindia region (odds ratio = 1.7; 95% confidence interval: 1.04–2.97); not intending to become pregnant for this pregnancy; belonging to a poor household; and having made 3, 4 or more ANC visits.

**Conclusion:**

This study reports a low proportion of women receiving the full package of ANC.

**Contribution:**

In order to improve this indicator, greater efforts need to be made in certain regions of the country to target pregnant women who achieve fewer ANC visits, carry pregnancies that were not intentional or belong to poorer households.

## Introduction

Worldwide, 33 women die every hour from causes related to pregnancy, childbirth or postpartum.^1^ Since 2016, the World Health Organization (WHO) has been recommending focused antenatal care (FANC) for all pregnant women at antenatal clinics.^1,2^ Focused antenatal care enables serological screening for syphilis, prevention of malaria, anti-tetanus vaccination, prevention of mother-to-child transmission of HIV and detection of malnutrition. It consists of measuring – during antenatal care (ANC) – blood pressure, weight, height, uterine height, abdominal circumference, auscultating foetal sounds, assessing the physical condition, administering tetanus vaccine, providing sulphadoxine–pyrimethamine (SP) and long-acting insecticide-treated mosquito nets to prevent malaria, and providing ferric folic acid (FFA). These services help to monitor the progress of the pregnancy and prepare the woman to accept medical assistance during childbirth.^1,2^

However, the provision of maternal health services to pregnant women by healthcare providers in low-income countries still leaves much to be desired in terms of both quality of care and effective coverage of services.^3^ A study in 10 low-income countries in Asia and Africa reported that although a proportion of pregnant women reported having had four or more ANC visits, the care provided did not meet the standards of quality required.^3^

In Africa, several studies have reported on FANC; for example, in Mozambique, it was reported in 2019 that the majority of women reported having an ANC visit and that only 13% of them received quality services during these visits.^4^ In 2016 in Kenya, Chorongo et al. reported that 3 out of 10 women had used FANC services.^2^ Similarly, in a district of Malawi in 2017, only 8% of women who came for initial visits received FANC.^5^

There are several reasons for this FANC coverage. A study carried out in the context of limited resources reported that beliefs, traditions, customs and norms and the misallocation of resources were associated with the provision of respectful, quality maternal health services to women.^6^ Chorongo et al. pointed out that in Kenya, religion, women’s lack of knowledge about the components of FANC, long waiting times and unavailability of services were associated with non-receipt of FANC.^2^ As in Nigeria in 2023, Nwabueze et al. reported late onset of ANC, long waiting times and overcrowded facilities as factors influencing the provision of FANC,^7^ and Hussen et al. in 2022 in Ethiopia, reported poor infrastructure, inadequate skilled personnel, stockouts of consumables and non-functioning basic emergency obstetric care facilities as factors in poor quality maternal health services in the least developed countries.^8^ Konlan et al. in Ghana also reported that long distances to health facilities were one of the factors negatively influencing receipt of FANC by pregnant women.^9^

In 2011, Fores et al. in Ethiopia reported that women whose spouses were present during ANC were more likely to undergo urine and blood tests.^10^

In Guinea, FANC services are offered to pregnant women at the time of their consultation.^11^ However, little is documented about the provision of these services at either national level or regional level. Indeed, studies reporting on ANC focused on the number of ANC visits and not on the use of the services contained in the ANC package.^12,13,14^ As a result, aspects of the FANC such as the level of use of the various services in the package, the proportion of women covered by the full package and the factors favouring the use of the full ANC package remain to be documented. This study therefore aims to fill this knowledge gap by analysing the use of FANC services in Guinea, using data from the Demographic and Health Survey (DHS) 2018. The results of this study would help guide ANC policies and practices in Guinea. The objective of this study was to determine the frequency and factors associated with the use of FANC services (blood pressure measuring, urine and blood sampling, receipt of FFA, deworming and SP tablets) among women attending at least one ANC visit in Guinea between 2016 and 2018.

## Research methods and design

### Study design

This was a secondary analysis of data from Guinea DHS 2018. The DHS is a nationally representative cross-sectional household survey.

### Study setting

Guinea is located in West Africa and had an estimated population of 13 million in 2014, 51.5% of whom were women.^15^ The majority of the population live in rural areas and are below the poverty line. The country comprises 33 prefectures organised into 7 administrative regions and 5 special communes forming the special region of Conakry. The government health system is organised into three levels: the primary level, comprising 407 rural and urban health centres, 26 district hospitals and 9 communal medical centres; the secondary level, comprising 8 regional hospitals; and the tertiary level, comprising 3 national hospitals.^16^

In general, 33% of women have their first birth before reaching the age of 18 years, and almost 30% of antenatal visits are initiated at the 4th month of pregnancy; 8 out of 10 women attended one ANC visit during pregnancy, but only 35% achieve four ANC visits.^16^ In terms of reproductive health, the total fertility rate is 4.8 children per woman. Men and women want an average of 5.4 and 7.1 children, respectively.^17^

### Study population

The study population included all women aged 15–49 years with the most recent live birth, attending at least one ANC visit for that birth, in the last 2 years preceding the Guinea DHS 2018.

### Study variables

#### Dependent variable

Our variable of interest was the use of all FANC services. This was a composite variable defined from the FANC service variables reported in the DHS 2018. They include blood pressure measurement, urine and blood sample collection, administration of FFA or dewormer and SP tablets. The ‘use of all FANC services’ was recoded as 1 ‘Yes’ if the woman was offered all of the single services listed; it was recoded as 0 ‘No’ if the woman did not receive one or more of these services.

#### Independent variables

Our independent variables were socio-demographic (age, level of education, marital status, socio-economic level, profession, residence, administrative region), desire for pregnancy and distance between home and health facility.

### Data analysis

The characteristics of the study population were summarised as percentages with 95% confidence intervals (CI). Univariate and multivariate logistic regressions were used to identify factors associated with the use of FANC services. The study sample was weighted before analyses. All variables with a *p*-value ≤ 0.20 were included in the logistic regression model. The unadjusted and adjusted odds ratios (OR) were derived. The significance level was set at 5% with a 95% CI. Data were processed and analysed using Stata 17 software (Stata Corp, College Station, Texas, United States [US]).

### Ethical considerations

This study followed all ethical standards for research without direct contact with human or animal subjects.

## Results

### Socio-demographic characteristics of the sample

A total of 2634 women (weighted number) were included in this study. Women aged between 25 years and 34 years were the most represented (42.3%). More than half (71.8%) had no formal education (Table 1). The majority (93.9%) were married and 43.7% had had 2 to 4 births.

**TABLE 1:** Socio-demographic characteristics of women having attended at least one antenatal care visit for their most recent live birth, 2018 Demographic and Health Survey (*N* = 2634)§.

Variables	*n*	%
**Age group during pregnancy (years)**		
15–19	499	19.4
20–24	625	23.9
25–29	671	25.2
30–34	451	17.1
35 or more	388	14.5
**Education level**		
No formal education	1898	71.8
Primary	345	13.0
Secondary	332	12.7
Higher	59	2.6
**Profession**		
Housewife	750	27.3
Trader or shopkeeper	57	2.4
Farmer	26	1.0
Service provider†	1125	4.4
Other professions‡	676	25.0
**Marital status**		
Unmarried	158	6.1
Married	2476	93.9
**Residence area**		
Urban	819	31.6
Rural	1815	68.4
**Administrative region**		
Boké	369	10.5
Conakry	239	12.2
Faranah	366	10.8
Kankan	450	19.1
Kindia	372	16.0
Labé	308	10.6
Mamou	246	7.0
N’Zérékoré	284	14.0
**Parity during ANC**		
No birth	515	19.9
1 previous birth	504	19.1
2 to 4 previous births	1148	43.7
5 or more previous births	467	17.3
**Household wealth index**		
Poor	1140	43.1
Medium	521	19.6
Rich	973	37.3
**Distance to the health facility perceived as a problem**		
Yes	1221	44.3
No	1413	56.7
**Desired this pregnancy**		
Yes	2196	83.3
No	438	16.7

ANC, antenatal care.

† , Service provider;

‡ , Other professions;

§ , Weighted number.

### Antenatal care coverage per number of visits

**FIGURE 1:**
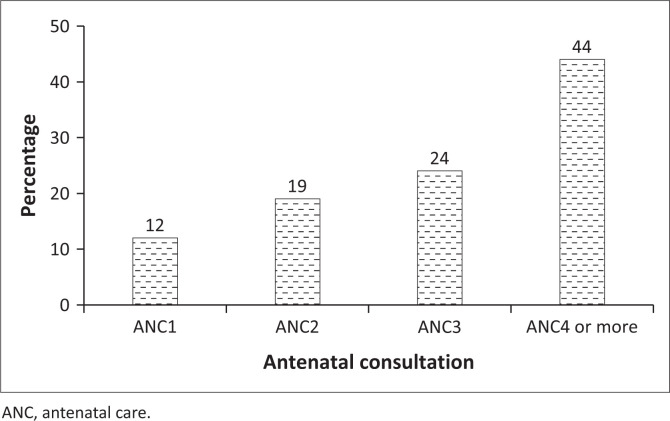
Antenatal care coverage by number of visits for the most recent births among women who attended at least one antenatal care visit, 2018 Demographic and Health Survey, Guinea.

Among women who had undergone ANC between 2016 and 2018, 12% had completed only one ANC visit; 19% and 24% had completed only 2 and 3 ANC visits, respectively (Figure 1). Those who had completed 4 or more ANC visits accounted for 44%.

### Use of focused antenatal care services

**FIGURE 2:**
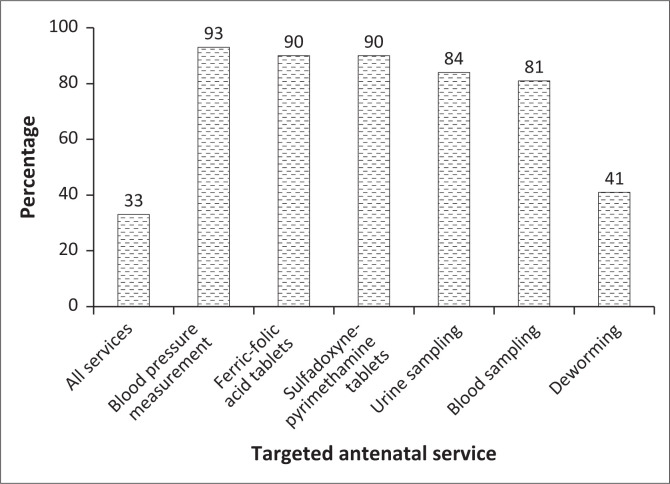
Uptake of focused antenatal care services for the most recent live birth among women who achieved at least one antenatal care visit, 2018 Demographic and Health Survey, Guinea.

Only a third of the women (33%) who had undergone ANC received all FANC services reported in this survey (Figure 2). Blood pressure measurement was the most used service (93%) and the least used service was deworming (42%).

### Factors associated with the use of the full package of antenatal care services

In the univariate analysis, the factors associated with the use of all FANC services were education level, residence area, administrative region, household wealth index, number of ANC visits and desire for pregnancy.

However, with the multivariate regression, the factors associated with the use of the package of ANC services were administrative region, number of ANC visits, household wealth index and desire for pregnancy (Table 2).

**TABLE 2:** Univariate and multivariate analyses of factors associated with the use of all focused antenatal care services among 15–49 year-old women for their most recent live birth during the last 24 months that preceded the 2018 Demographic and Health Survey, Guinea (*N* = 2634).

Variables	Received all FANC services (yes)						
	Effective	Crude OR	95% CI	*p*	Adjusted OR	95% CI	*p*
**Age group during pregnancy (years)**							
15–19	499	0.9	0.65–1.22	0.473	-	-	-
20–24	625	1.1	0.85–1.42	0.464	-	-	-
25–29	671	1.0	0.77–1.33	0.924	-	-	-
30–34	Ref	-	-	-	-	-	-
35 or more	273	0.9	0.67–1.26	0.606	-	-	-
**Profession**							
Housewife	750	0.9	0.68–1.12	0.289	0.8	0.60–1.01	0.060
Trader or shopkeeper	57	1.4	0.71–2.69	0.327	0.8	0.42–1.78	0.689
Farmer	26	1.9	0.67–5.05	0.227	1.2	0.42–3.25	0.765
Service provider	1125	Ref	-	-	Ref	-	-
Other professions	676	1.4	1.02–1.79	0.032	1.0	0.76–1.62	0.996
**Education level**							
No formal education	1898	0.5	0.26–0.90	0.022	0.8	0.37–1.67	0.531
Primary	345	0.7	0.33–1.26	0.205	0.9	0.40–1.91	0.728
Secondary	332	0.8	0.40–1.44	0.411	0.8	0.39–1.70	0.592
Higher	59	Ref	-	-	Ref	-	-
**Residence area**							
Urban	819	Ref	-	-	Ref	-	-
Rural	1815	0.5†	0.34–0.59	0.001	0.7†	0.70–0.47	0.065
**Marital status**							
Unmarried	158	1.2	0.84–1.79	0.274	-	-	-
Married	2476	Ref	-	-	-	-	-
**Administrative region**							
Boké	369	0.3†	0.17–0.54	0.001	0.6	0.35–1.21	0.173
Conakry	239	Ref	-	-	Ref	-	-
Faranah	366	0.7	0.40–1.09	0.113	1.6	0.90–2.77	0.112
Kankan	450	0.5	0.29–0.81	0.006	1.0	0.54–1.69	0.877
Kindia	372	1.1	0.66–1.70	0.000	1.8†	1.08–3.05	0.025
Labé	308	0.3	0.17–0.49	0.796	0.6	0.35–1.11	0.107
Mamou	246	0.6	0.37–1.02	0.065	1.3	0.73–2.46	0.350
N’Zérékoré	284	1.1	0.66–1.87	0.001	2.8†	1.51–5.13	0.001
**Distance to the health facility perceived as a problem**							
Yes	1221	1.0	0.75–1.22	0.736	-	-	-
No	1413	Ref	-	-	-	-	-
**Number of ANC visits achieved**							
1 visit	324	-	-	-	Ref	-	-
2 visits	502	1.4	0.91–2.22	0.113	1.3	0.82–1.92	0.296
3 visits	660	2.7†	1.78–4.21	0.001	2.4†	1.63–3.65	0.001
4 visits or more	1148	4.3†	2.81–6.54	0.001	3.5†	2.38–5.29	0.001
**Household wealth index**							
Poor	1140	0.4†	0.32–0.56	0.001	0.6†	0.44–0.85	0.001
Medium	521	0.6†	0.47–0.81	0.001	0.8	0.59–1.13	0.217
Rich	973	Ref	-	-	Ref	-	-
**Parity during ANC**							
No birth	515	Ref	-	-	-	-	-
1 previous birth	504	1.1	0.83–1.50	0.452	-	-	-
2–4 previous births	1148	1.0	0.78–1.29	0.942	-	-	-
5 or more previous births	467	0.8	0.62–1.15	0.295	-	-	-
**Desired this pregnancy**							
Yes	2196	Ref	-	-	Ref	-	-
No	438	0.8	0.60–1.02	0.081	0.7†	0.56–0.99	0.042

FANC, focused antenatal care; ANC, antenatal care; CI, confidence interval; OR, odds ratio; Ref, reference.

† , *p* < 0.05.

Women in the N’Zérékoré region were three times more likely to receive all FANC services (odds ratio [OR] = 3; 95% confidence interval [CI]: 1.51–5.13; *p* = 0.001) compared with those in Conakry (Table 2). Similarly, women in Kindia were 1.8 (OR = 1.7; 95% CI: 1.08–3.05; *p* = 0.025) times more likely to receive all FANC services than women in Conakry.

Women who had undergone three ANCs visits and those who had undergone four or more ANCs visits were 2.4 (OR = 2.4; 95% CI: 1.63–3.65; *p* = 0.000) and 3.5 (OR = 3.5; 95% CI: 2.38–5.29; *p* = 0.000) times more likely to receive all FANC services, respectively, compared with those who had received one visit (Table 2).

Compared with rich women, poor women were 40% (OR = 0.6; 95% CI: 0.44 – 0.85; *p* = 0.001) less likely to receive all FANC services (Table 2).

Women with unintended pregnancies were 30% (OR = 0.7; 95% CI: 0.56 – 0.99; *p* = 0.042) less likely to use all FANC services compared with those with intended pregnancies (Table 2).

## Discussion

This study shows that between 2016 and 2018, one-third of women undergoing ANC received all FANC services; blood pressure was measured in almost all women; however, less than half of women received deworming tablets. Women who were more likely to receive all FANC services were those living in the Kindia or N’Zérékoré regions and who had made three, four or more ANC visits. However, those who had not desired to get pregnant and belonged to a poor household were less likely to receive all FANC services.

This study reveals that despite the use of ANC, women are not receiving all the services recommended for ANC. This underlines the challenge of access to services among women undergoing ANC (33%). Indicators found in our study are better than those reported in Mozambique and Ghana in 2019 and Ethiopia in 2021, where only 13%, 12.6% and 6.1% of women had received quality ANC, respectively.^4,18,19^ However, our results would differ from those found by Radovith et al. in India, where 9 out of 10 women would have benefited from all FANC services. Our results can be explained by different factors. Firstly, it could be because of the lack or shortage of inputs (reagents, strips) and drugs (FFA, SP and dewormer) and the lack of equipment (tensiometer, microscope) in the health facilities. Secondly, it could be linked to non-compliance with ANC service standards and procedures by health staff. Thirdly, cultural factors influencing women’s perception of these services could lead them to refuse certain services contained in the FANC. Fourthly, logistical factors could compromise the timely delivery of inputs and medicines for ANC, leading to their stockouts.

Under-use of the services contained in the ANC package could indeed expose women, even if they do undergo ANC, to the risk of illnesses such as anaemia (e.g., if they do not take FFA, SP tablets and deworming tablets), malaria and parasitosis. Qualitative studies would be needed to better explore the realities associated with the shortage of inputs and medicines, as well as the adherence of healthcare providers to the guidelines for the provision of ANC services; this would enable a better understanding of the influence of these realities on women’s use of FANC services.

Our results also show that less than half of the women who underwent at least one ANC visit received deworming medicines. This indicator is lower than the one reported in 2014 in Nepal.^20^ This low rate of deworming could be partly because of stock-out or low supply of deworming medicines in health facilities. It is therefore necessary to make deworming medicines available in health facilities.

In our study, almost one out of every two women attending ANC was from a poor household. Women from richer households were more likely to use all FANC services than women from poorer households. These results are comparable to those found in Ghana in 2018 where wealth had a positive association with the use of ANC services.^21^ The low uptake of FANC services by these poor women can be explained by certain staff attitudes, such as discrimination against poorer women and the high cost of certain examinations (blood and urine tests).^22,23^ This calls for the need not only to promote among health staffs the provision of respectful and equitable ANC services but also to ensure that ANC services are offered free of charge.

### Limitations and strengths

The main limitation is that the data analysed being secondary, it was not possible to take into account in the analysis the other WHO-recommended FANC services, given these information pieces were not collected during the DHS. The services on which information was not collected were weight measurement, abdominal circumference and the receipt of various vaccines. In addition, possible reporting and recall bias could be a limitation to this study; also, the DHS cannot determine a cause-and-effect relationship because data are collected retrospectively. However, the national representativeness of DHS is a major strength as such study provides useful information to guide national and sub-national FANC programming and stimulate further study.

## Conclusion

This study shows that, between 2016 and 2018, only one-third of women who had at least one ANC visit in Guinea had received all FANC services. Deworming was the least offered service. Factors associated with receiving all services were region, poverty, unwanted pregnancy and having three and four or more ANC visits.

In the Guinean context, there is a need to prevent health facilities from running out of ANC drugs, particularly for deworming, and to provide these facilities with essential equipment (tensiometer, microscope, laboratory reagents). It is also important to continuously disseminate ANC standards and procedures to health providers, so that they can provide comprehensive, high-quality ANC services.
